# Nurse-based secondary preventive follow-up by telephone reduced recurrence of cardiovascular events: a randomised controlled trial

**DOI:** 10.1038/s41598-021-94892-0

**Published:** 2021-08-02

**Authors:** Anna-Lotta Irewall, Anders Ulvenstam, Anna Graipe, Joachim Ögren, Thomas Mooe

**Affiliations:** Department of Public Health and Clinical Medicine, Östersund, Umeå University, Umeå, Sweden

**Keywords:** Cardiology, Stroke, Risk factors, Preventive medicine, Outcomes research, Cardiovascular diseases, Hypertension

## Abstract

Enhanced follow-up is needed to improve the results of secondary preventive care in patients with established cardiovascular disease. We examined the effect of long-term, nurse-based, secondary preventive follow-up by telephone on the recurrence of cardiovascular events. Open, randomised, controlled trial with two parallel groups. Between 1 January 2010 and 31 December 2014, consecutive patients (n = 1890) admitted to hospital due to stroke, transient ischaemic attack (TIA), or acute coronary syndrome (ACS) were included. Participants were randomised (1:1) to nurse-based telephone follow-up (intervention, n = 944) or usual care (control, n = 946) and followed until 31 December 2017. The primary endpoint was a composite of stroke, myocardial infarction, cardiac revascularisation, and cardiovascular death. The individual components of the primary endpoint, TIA, and all-cause mortality were analysed as secondary endpoints. The assessment of outcome events was blinded to study group assignment. After a mean follow-up of 4.5 years, 22.7% (n = 214) of patients in the intervention group and 27.1% (n = 256) in the control group reached the primary composite endpoint (HR 0.81, 95% CI 0.68–0.97; ARR 4.4%, 95% CI 0.5–8.3). Secondary endpoints did not differ significantly between groups. Nurse-based secondary preventive follow-up by telephone reduced the recurrence of cardiovascular events during long-term follow-up.

## Introduction

Patients with previous acute manifestations of cardiovascular or cerebrovascular disease are at high risk of recurring cardiovascular events^1–3^. Due to the ageing population and decreasing case fatality, this high-risk group is growing in absolute numbers and secondary prevention is a lifelong perspective^4,5^. This makes effective implementation of secondary preventive measures in clinical practice both important and challenging. Reports of insufficient achievements in terms of modifiable risk factor control have been published for more than 20 years^6–9^. This may reflect the fact that evidence guiding the implementation of enhanced, long-term, and cost-effective secondary preventive follow-up on the population level is still scarce.

Different secondary prevention programmes have been evaluated in randomised controlled trials (RCTs)^10–22^, but most studies have had a short-term perspective^11,15,17,18,20,21^ and small study samples^11,15–17,19,22^, and few have evaluated the effect on the recurrence of cardiovascular events^10,12–14,17,20^. To make interventions broadly feasible in clinical practice, there is good reason to keep follow-up procedures as simple as possible. As high-intensity follow-up has been a common feature of both positive and neutral trials^10,11,13,16,17,19,21,22^ no firm conclusions can be drawn about the required frequency intervals of follow-ups.

The Nurse-based Age-independent Intervention to Limit Evolution of Disease (NAILED) trial was an interventional RCT initiated in 2010 to investigate whether improvements in cardiovascular risk and outcome can be achieved through a structured, telephone-based follow-up programme including life style counselling, repeated risk factor assessment, and adjustment of pharmacological treatment to meet treatment target levels. Results regarding risk factor control after 12 and 36 months of follow-up were published previously and showed favourable outcomes in terms of lower blood pressure and low density lipoprotein cholesterol (LDL-C) levels in the intervention group compared to the control group^23–26^.

In the present cardiovascular outcome study, NAILED-CV, we hypothesised that long-term secondary preventive follow-up according to the NAILED intervention programme would decrease the risk of stroke, myocardial infarction (MI), cardiac revascularisation, and cardiovascular death.

## Methods

### Study design

The NAILED-CV trial was an interventional, single-centre, open RCT with two parallel groups. The research protocol is available as a supplement (Reasearch Protocol).

### Participants

We identified consecutive patients hospitalised due to acute MI, unstable angina (UA), ischaemic or haemorrhagic stroke (excluding subarachnoid haemorrhage, SAH), or transient ischemic attack (TIA). The study took a pragmatic approach and strived to include as unselected a study sample as possible. However, the study intervention follow-up required the participants to be physically and cognitively able to communicate by telephone and to receive information and health instructions by this medium. Consequently, patients with aphasia or severely impaired hearing or cognition (i.e., dementia) were excluded. In addition, patients in whom intensive secondary preventive measures were considered inept due to severely impaired health, often with short remaining life expectancy, were excluded. This included patients severely disabled due to stroke, heart disease, or other conditions, including terminal cancer. In addition, we excluded participants of concurrent, incompatible clinical trials.

### Setting

The inclusion period ran from 1 January 2010 to 31 December 2014. During the last year of inclusion, only patients with MI or UA as the qualifying event were included. The intervention follow-up and follow-up of outcome events was performed until 31 December 2017. Participants who moved out of the county could no longer be followed for outcome events through the medical records and were considered lost to follow-up. For these participants, follow-up of outcome events terminated at the date of migration or, when not available, the date of the last documented medical contact.

Participants were recruited at Östersund Hospital, which is the only hospital in the county of Jämtland-Härjedalen and, thus, the only referral centre in the county for patients with suspected MI, stroke, or TIA. Patients with these diagnoses are generally referred and treated in-hospital, with the exception of some patients in terminal care. At the beginning of the study, the county had approximately 126 500 inhabitants, 35.0% of which lived in the centrally located city of Östersund, and the remaining in villages and the more sparsely populated surroundings. Primary health care was provided by 28 primary health care centres. During the first half of the study period, all patients in need of invasive cardiac revascularisation (coronary artery bypass grafting, CABG, or percutaneous coronary intervention, PCI) were referred to the University Hospital of Northern Sweden, Umeå. Elective invasive coronary angiography was performed in Östersund during the entire study period. In 2015, the county of Jämtland established a 24/7 primary PCI network, after which all PCI procedures were performed at Östersund Hospital.

### Randomisation

At inclusion, participants were randomly assigned to the intervention or control group (1:1). The randomised allocation sequences were computer-generated in blocks of four and put into sequentially numbered, opaque, sealed envelopes (managed by T.M.). For participants with stroke or TIA as the qualifying event, the randomisation was stratified for sex and degree of disability (modified Rankin scale < 3 or ≥ 3) close to hospital discharge. The randomisation of participants with MI or UA was stratified for sex and the qualifying event. The resulting group allocation was not blinded to the participants, study team, or other caregivers. The process of enrolment and random group assignment was performed by study nurses.

### Intervention follow-up

The intervention consisted of a secondary preventive follow-up programme in which study nurses systematically performed telephone-based follow-up of cardiovascular risk factors and pharmacological treatment. Before each follow-up call, the blood pressure and blood lipids were measured. During telephone counselling, the nurses interviewed the patients about adherence and persistence to pharmacological treatment and life style-related matters. They encouraged physical activity (a minimum 30 min/day, 5 days/week), smoking cessation, and a diet in accordance with the recommendations from the Swedish Food Agency. Briefly, the latter included general recommendations to increase the intake of fruits, vegetables, and fibre and to decrease the intake of saturated fats in favour of unsaturated fats. When participants did not meet the treatment target levels for blood pressure or LDL-C, the study nurse consulted a study physician for evaluation and adjustment of the pharmacological treatment. All pharmacological adjustments were individualised and not restricted to any fixed algorithm or protocol. The treatment target level for blood pressure was < 140/90 mmHg throughout the study period. The corresponding level for LDL-C was < 2.5 mmol/l when the study began but changed during the course of the study to comply with updates of the local guidelines. On 31 March 2013, the level was lowered to < 1.8 mmol/l for patients with diabetes, and this treatment target was adopted for all patients with established cardiovascular disease (i.e., all study participants) from 1 January 2017. The first follow-up occurred 1 month after discharge, and then yearly thereafter until the intervention follow-up was terminated. Adjustments to pharmacological treatment were evaluated through a new contact after 4 weeks. When necessary, further adjustments were made and the procedure repeated until the treatment target was reached or no further adjustments were considered possible.

### Secondary preventive follow-up in the control group

Participants in the control group received secondary preventive follow-up in accordance with local standard procedures. Secondary preventive treatment was generally initiated in hospital. Patients with stroke or TIA were referred to their general practitioner (GP) after discharge. Most patients with MI or UA had a follow-up visit with a cardiology nurse after 1 month and with a cardiologist after 2–3 months.

For all patients, their GP at the primary health care centre held primary responsibility for the long-term secondary preventive follow-up.

### Outcomes

The primary outcome was a composite of the first occurrence of MI, stroke, cardiac revascularisation or cardiovascular death. Secondary outcome measures included the individual components of the primary outcome, all-cause mortality, and TIA. Our definition of MI was based on the 3^rd^ universal definition, and only type 1 MIs were counted as outcome events. Stroke was defined as an acute episode of focal or global neurological dysfunction caused by cerebral infarction or spontaneous haemorrhage, excluding SAH. Episodes of focal cerebral dysfunction without evidence of brain infarction and with symptoms resolving within 24 h were counted as TIA. Cardiac revascularisation included PCI and CABG, regardless of indication. Events of stroke, TIA, and MI had to be evaluated at the hospital to be counted as outcome events. Cause of death was classified as cardiovascular or non-cardiovascular based on the underlying cause of death, which was determined based on all available information in the medical record, including the death certificate and post-mortem report (when performed). An MI or stroke followed by death within 30 days was considered fatal and included in the primary composite outcome as a cardiovascular death. A detailed description of event definitions is available in Supplementary Methods 1.

### Data collection

Baseline characteristics were collected in-hospital during hospitalisation for the qualifying event by study nurses through participant interviews and a review of the medical records.

Identification and review of potential outcome events was performed by four medical doctors who were part of the study team. The review process followed a standardised work-flow routine in which the reviewers were unaware of the study group allocation of participants and events were strictly evaluated according to the study outcome definitions. Each reviewer worked with their assigned cases independently, but consecutive meetings were held to discuss difficult cases as well as more general matters concerning appliance of the event definition.

To identify potential outcome events, all discharge records for hospitalisations at the Department of Internal Medicine during the study follow-up period were obtained and scrutinised. This included hospitalisation at the stroke unit and the cardiology unit, as well as other internal medicine wards. To identify events occurring at other hospital departments, we used the hospital in-patient register to search for relevant discharge diagnoses (Supplementary Methods 2). Events of cardiac revascularisation were identified through the Swedish Coronary Angiography and Angioplasty Registry (SCAAR). For all potential events identified through these two registries, the medical records were reviewed to confirm accordance with the respective outcome definition and preclude duplicate registrations. For stroke, TIA, and MI, the date of hospitalisation was set as the event date.

### Sample size

We planned for study groups of approximately 1000 participants to have 80% power to detect an absolute risk reduction for the primary outcome of 6% (two-sided alpha 0.05), assuming an incidence of approximately 40% in the standard treatment group during a mean follow-up of 4 years. The assumed incidence rate was a conservative estimate originally based on unpublished 2-year outcome data from the KAPRIS project, a non-randomized, secondary preventive, interventional study with similar inclusion criteria conducted in the same geographical region between 2007 and 2009. The ARR was an arbitrary estimate based on what we believed would be of clinical significance and also a difference large enough to have the potential to change the prevailing practice.

During the course of the study, the inclusion period was extended to include a larger study sample. The purpose was to enable sub studies of certain risk groups, but it was also an adjustment to meet the general decline in cardiovascular event rate reported by contemporary studies.

### Statistical analysis

We conducted all analyses in accordance with the intention-to-treat principle. The number of cases with missing data was small and reported for each variable separately. We did not use imputation. Baseline characteristics are presented as median values with interquartile ranges (IQR) for continuoues variables and as proportions (percentages) for categorical variables. For between-group comparisons, we used the Mann–Whitney U-test, chi-squared test, or Fischer’s exact test as appropriate. All tests were two-sided and significance determined at an alpha level of 0.05. Cumulative incidence of the primary and secondary outcomes was presented using Kaplan–Meier survival analysis with log rank test for between-group comparisons. The day of discharge was set as day 0. Univariate Cox proportional hazards regression was used to calculate hazard ratios for outcome events and the result was also presented as absolute risk reduction (ARR) and numbers needed to treat (NNT).

Baseline characteristics and the occurrence of outcome events were compared between subgroups defined by sex and qualifying event. The same statistical methods were used with the addition of an interaction analysis in which randomised group allocation and the respective subgroups were included as variables in Cox proportional hazards models, both individually and as interaction variables. All analyses were performed using SPSS software, version 24.0.

### Trial registration

The NAILED-CV trial is registered in the ISRCTN registry (ISRCTN30433343). The strict ICMJE requirement of prospective registration of clinical trials came to our attention when recruitment had already begun. Therefore, the study was retrospectively registered (4 December 2014).

### Ethics approval

The study was originally approved by the Regional Ethics Review Board, Umeå, Sweden, on 28 October 2009 (ref: Dnr 09-142M). An amendment mainly concerning an extended follow-up period was passed on 10 June 2013 (ref: Dnr 13-204-32M). The study was performed in accordance with the Declaration of Helsinki and all participants provided informed written consent of participation.

## Results

**Figure 1:**
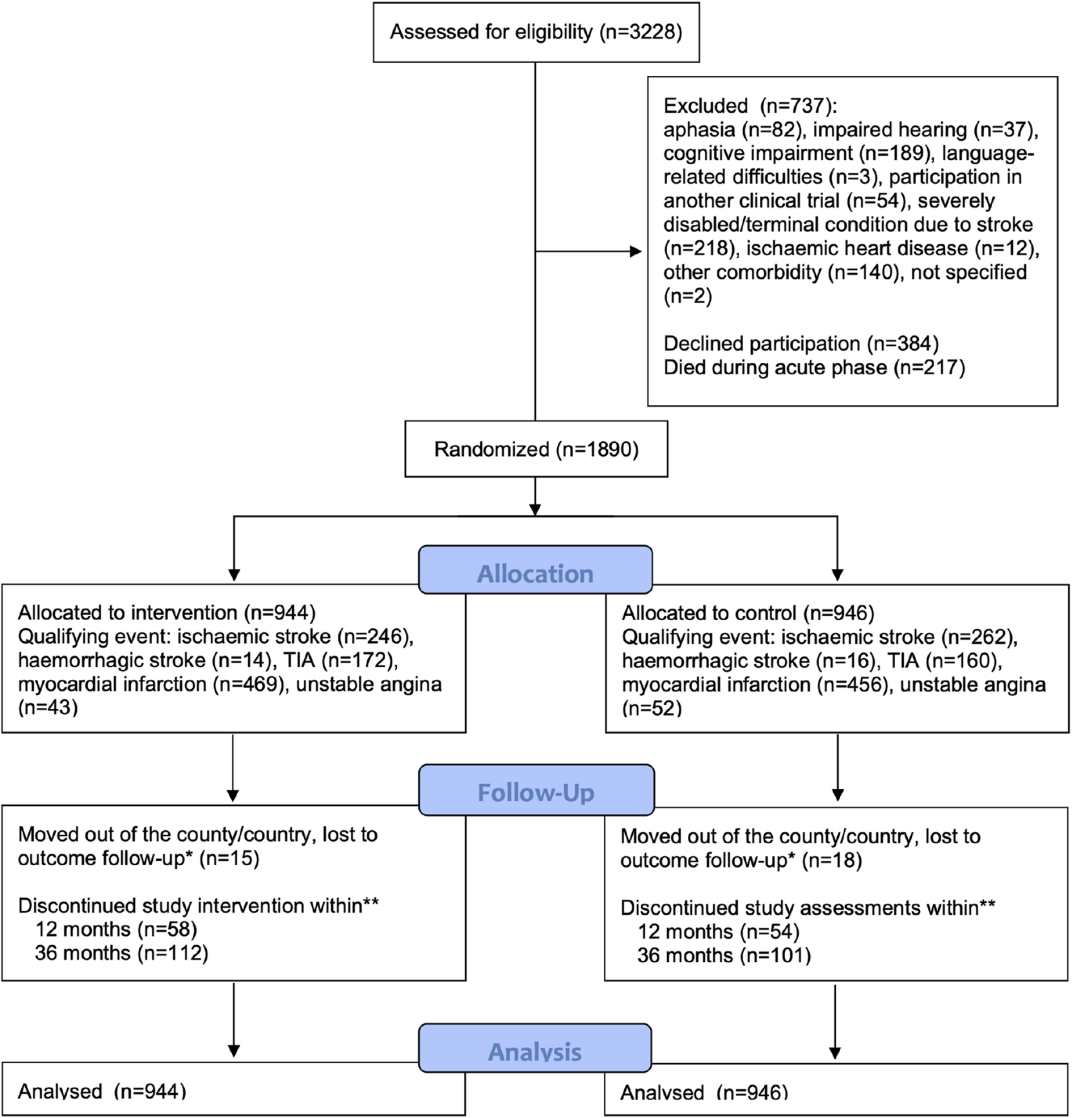
Study flow chart. *Patients who moved were censored from the outcome analysis at the date they moved or, when unavailable, the date of the last documented medical contact. **Cumulative count.

Between 1 January 2010 and 31 December 2014, 3228 consecutive patients with acute stroke, TIA, MI, or UA were identified. Among those who survived the acute phase (n = 3011), 1890 patients (62.8%) were included in the follow-up study and randomly assigned to the intervention group (n = 944) or control group (n = 946). Patients included in the study constituted 83.1% of all patients considered physically and cognitively able to participate in the telephone-based follow-up. The flow of participants is illustrated in Fig. 1.

The median age of the participants was 71.0 (IQR 63.1–78.7) years, and 36.5% (n = 689) were women. MI (n = 925, 48.9%) was the most common qualifying event, followed by stroke (n = 538, 28.5%), TIA (n = 332, 17.6%), and UA (n = 95, 5.0%). Baseline characteristics were well balanced between the randomised groups, except for a slightly higher proportion of participants with low education level (≤ 10 years of formal education) in the control group (*p* = 0.040, Table 1).

**Table 1:** Baseline characteristics of the study population.

	Intervention	Control
N (%)	944 (49.9)	946 (50.1)
Women	346 (36.7)	343 (36.3)
Age, years	70.6 (62.6–79.0)	71.4 (63.7–78.5)
Low education level*	451 (47.9)	496 (52.6)
BMI^†^	26.6 (24.0–29.6)	26.5 (23.9–29.5)
eGFR, ml/min^‡^	81.1 (66.6–92.2)	82.0 (65.5–91.7)
Systolic BP, mmHg^§^	140.0 (123.0–152.0)	138.0 (121.0–151.0)
Diastolic BP, mmHg**	79.0 (70.0–85.0)	78.0 (70.0–85.0)
LDL-C, mmol/l^††^	3.2 (2.4–3.9)	3.1 (2.4–3.8)
mRS > 2^‡‡^	68 (7.2)	69 (7.3)
Current/former smoker^§§^	540 (57.3)	538 (56.9)
Atrial fibrillation	155 (16.4)	144 (15.2)
Ischaemic heart disease	152 (16.1)	174 (18.4)
Peripheral artery disease	23 (2.4)	22 (2.3)
Diabetes	168 (17.8)	182 (19.2)
CKD (GFR < 60 ml/min)***	159 (17.0)	183 (19.4)
Congestive heart failure	39 (4.1)	34 (3.6)
Hypertension	538 (57.0)	550 (58.1)
Previous stroke	86 (9.1)	84 (8.9)
Previous TIA	37 (3.9)	32 (3.4)
Antihypertensive treatment	829 (87.8)	829 (87.6)
1 drug	198 (21.0)	216 (22.8)
2 drugs	347 (36.8)	330 (34.9)
≥ 3 drugs	284 (30.1)	283 (29.9)
Lipid-lowering agent	796 (84.3)	804 (85.0)
Antiplatelet drug	835 (88.5)	843 (89.1)
Warfarin	115 (12.2)	97 (10.3)

Data are given as N (%) or median (interquartile range). No significant differences were present except a higher proportion of participants with low education in the control group (*p* = 0.040). Low education level was defined as no more than 10 years of formal education. Drug treatment variables refer to treatment at discharge. BMI, body mass index; eGFR, estimated glomerular filtration rate; BP, blood pressure; LDL-C, low-density lipoprotein cholesterol; CKD, chronic kidney dysfunction; TIA, transient ischemic attack.

*Missing values for 3 control group participants and 2 intervention group participants.

^†^Missing values for 2 control group participants and 1 intervention group participant.

^‡^Missing values for 3 control group participants and 9 intervention group participants.

^§^Missing values for 11 control group participants and 18 intervention group participants.

**Missing values for 4 control group participants and 3 intervention group participants.

^††^Missing values for 34 control group participants and 39 intervention group participants.

^‡‡^Missing values for 4 control group participants and 2 intervention group participants.

^§§^Missing value for 1 intervention group participant.

***Missing values for 3 control group participants and 9 intervention group participants.

Compared to patients excluded from the study, participants were younger, included a higher proportion of men, and cardiovascular comorbidity and risk factors were generally less prevalent. Patients who declined participation had characteristics more similar to the excluded group than to the participants (Supplementary Table 1).

**Figure 2:**
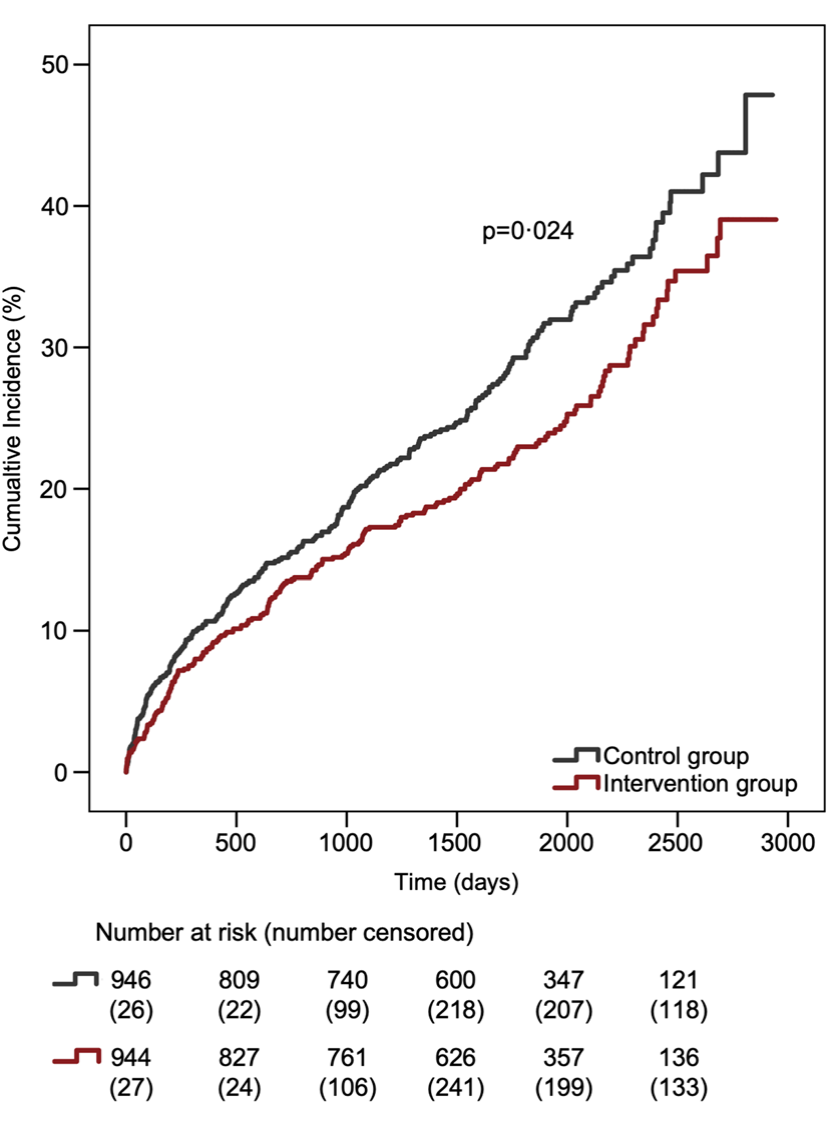
Cumulative incidence of the primary outcome. The primary outcome included non-fatal stroke, non-fatal myocardial infarction, cardiac revascularisation, and cardiovascular death.

During a mean follow-up of 4.5 years, a total of 470 patients (5.5% per year at risk) reached the primary composite endpoint, with a significantly lower incidence in the intervention group (HR 0.81, 95% CI 0.68–0.97) compared to the control group (Fig. 2).

In the intervention group, the primary endpoint occurred in 22.7% (n = 214) of participants, compared to 27.1% (n = 256) in the control group, resulting in an ARR of 4.4% (95% CI 0.5–8.3) and NNT of 22.7. Though cardiovascular death was similar between groups, all the other three components of the composite primary endpoint (stroke, MI, cardiovascular revascularisation) appeared to numerically favour the intervention group (Table 2), i.e. the observed difference in the primary endpoint was not driven by any specific endpoint component. No significant differences were seen for any of the secondary endpoints, although there might be a trend in favour of the intervention group regarding the occurrence of stroke (HR 0.76, 95% CI 0.56–1.02, *p* = 0.07).

**Table 2:** Primary and secondary endpoints.

	Intervention, N (%)	Control, N (%)	Absolute difference (%)	HR (95% CI)	*p* value
**Primary endpoint**					
Cardiovascular death, MI, stroke, or cardiac revascularisation*	214 (22.7)	256 (27.1)	− 4.4	0.81 (0.68–0.97)	0.02
Cardiovascular death	64 (29.9)	62 (24.2)			
MI (non-fatal)	38 (17.8)	54 (21.1)			
Stroke (non-fatal)	65 (30.4)	84 (32.8)			
Cardiac revascularisation	47 (22.0)	56 (21.9)			
**Secondary endpoints**					
All-cause mortality	211 (22.4)	220 (23.3)	− 0.9	0.94 (0.78–1.14)	0.54
Cardiovascular death	88 (9.3)	105 (11.1)	− 1.8	0.82 (0.62–1.09)	0.18
Myocardial infarction	57 (6.0)	73 (7.7)	− 1.7	0.77 (0.54–1.08)	0.13
STEMI	16 (28.1)	11 (15.1)			
NSTEMI	41 (71.9)	62 (84.9)			
Fatal within 30 days	10 (17.5)	10 (13.7)			
Cardiac revascularisation	70 (7.4)	79 (8.4)	− 1.0	0.87 (0.63–1.20)	0.40
PCI	56 (80.0)	61 (77.2)			
CABG	14 (20.0)	18 (22.8)			
Stroke	78 (8.3)	101 (10.7)	− 2.4	0.76 (0.56–1.02)	0.07
Ischaemic	74 (94.9)	87 (86.1)			
Haemorrhagic	3 (3.8)	12 (11.9)			
Undefined	1 (1.3)	2 (2.0)			
Fatal within 30 days	11 (14.1)	12 (11.9)			
TIA	31 (3.3)	36 (3.8)	− 0.5	0.85 (0.53–1.37)	0.50

The primary endpoint components, subtypes of the secondary endpoints, and fatality are presented as proportions (%) of the main outcome event for each group. MI, Myocardial infarction; STEMI, ST elevation myocardial infarction; NSTEMI, Non-ST elevation myocardial infarction; PCI, Percutaneous coronary intervention; CABG, Coronary artery bypass grafting; TIA, Transient ischemic attack.

*The first event to occur was counted.

Subgroup analysis of the primary endpoint showed very similar results across groups defined by the qualifying event (stroke/TIA vs. MI/UA), but did not reach statistical significance at this level. The weight of the different endpoint components differed between subgroups (Table 3). Among patients with MI or UA as the qualifying event, the difference between the randomised groups was driven mainly by a lower occurrence of new cardiac events in the intervention group. In contrast, a difference in the occurrence of new stroke events was predominant among stroke/TIA participants. Overall, stroke was the dominating component of the primary endpoint among stroke/TIA participants, whereas a more even distribution of events was seen among AMI/UA participants.

**Table 3:** Distribution of the primary endpoint and its components in subgroups defined by qualifying event and sex.

	Primary endpoint	Primary endpoint components			
		CV death	MI	Stroke	Revasc
**Intervention group**					
Myocardial infarction/unstable angina	117 (22.9)	31 (26.5)	30 (25.6)	21 (17.9)	35 (29.9)
Stroke/TIA	97 (22.5)	33 (34.0)	8 (8.2)	44 (45.4)	12 (12.4)
Women	81 (23.4)	29 (35.8)	13 (16.0)	30 (37.0)	9 (11.1)
Men	133 (22.2)	35 (26.3)	25 (18.8)	35 (26.3)	38 (28.6)
**Control group**					
Myocardial infarction/unstable angina	137 (27.0)	37 (27.0)	37 (27.0)	19 (13.9)	44 (32.1)
Stroke/TIA	119 (27.2)	25 (21.0)	17 (14.3)	65 (54.6)	12 (10.1)
Women	102 (29.7)	23 (22.5)	22 (21.6)	37 (36.3)	20 (19.6)
Men	154 (25.5)	39 (25.3)	32 (20.8)	47 (30.5)	36 (23.4)

Data are presented as N (%).

CV death, Cardiovascular death; MI, Myocardial infarction; Revasc, Cardiac revascularisation; TIA, Transient ischemic attack.

In subgroup analyses based on sex, the between group difference in incidence of the primary endpoint appeared to be more pronounced among women than men, but the interaction was not significant (*p* = 0.48). The results of subgroup analyses for secondary endpoints are available in Supplementary Table 2. Among participants with stroke/TIA as the qualifying event, the incidence of stroke during follow-up was lower in the intervention group compared to the control group (12.0% vs. 17.4%, HR 0.68, 95% CI 0.48–0.98), but the interaction was not significant (*p* = 0.253).

## Discussion

In the present study, the nurse-based, secondary preventive follow-up focused on improving modifiable risk factors resulted in a lower incidence of the composite of cardiovascular death, MI, stroke, and cardiac revascularisation during long-term follow-up. The benefits of the intervention appeared to be similar regardless of the qualifying event (stroke/TIA or ACS), although the result regarding the primary endpoint was not significant at the subgroup level. Secondary endpoints did not differ significantly between the intervention group and the control group, although there was a trend towards lower incidence of stroke in the intervention group, a trend driven by a difference in the subgroup with stroke/TIA as the qualifying event.

Several previous RCTs of enhanced secondary preventive follow-up have achieved improved risk factor levels^10,11,15,17–19,21,22^, but very few have been large enough to evaluate the effect on long-term recurrence of cardiovascular events^10,12,13^. In PREseAP (n = 1224), nurse-led follow-up every 4 months over 2.75 years did not significantly improve risk factor levels or recurrence of cardiovascular events at 3 years in a population of ischaemic heart disease/stroke/TIA/peripheral artery disease patients^12^. INSPiRE-TMS (n = 2098) evaluated a secondary preventive programme focused on risk factor management after acute stroke/TIA^10^. The programme was delivered as eight face-to-face appointments with a nurse and physician over 2 years. Though the intervention did improve risk factor levels at 3 years, it did not translate into any significant reduction of major cardiovascular events. Notably, the between-group difference in blood pressure and LDL-C levels at 3 years was larger in the NAILED trial than INSPiRE-TMS^23,26^. As the control groups achieved similar results between studies, this was primarily a result of better achievements in the NAILED intervention group. In the GOSPEL trial (n = 3241), 2-h multidisciplinary sessions delivered monthly for 6 months and then every half a year resulted in improved levels of physical activity and psychological stress, improved dietary habits, and a reduction in secondary cardiovascular outcome events after 3 years^13^. However, the results regarding the composite primary cardiovascular outcome were not significant and, compared to the NAILED-CV trial, the GOSPEL study sample had a much lower mean age, considerably lower incidence of cardiovascular events during follow-up overall, and the intervention was comparably high in resource demand.

The overall event rate in the NAILED-CV trial turned out lower than what was expected when the trial was originally designed. We believe that this was a natural consequence of the reduction in both cardiovascular mortality and the incidence of myocardial infarction and stroke seen in Sweden^27,28^ and other countries^29^ during the last decades. The event rates in clinical trials have also decreased, requiring larger study samples for proper outcome evaluation^30^. Through extension of the inclusion period we increased our study sample, which increased the possibility to detect a clinically relevant difference between groups (avoiding a type 2 error).

### Mechanisms

Previous publications from the NAILED trial have shown that the intervention group achieved significantly lower blood pressure and LDL-C levels than the control group at 12 months^24,25^, and that the gap between the groups continued to increase over the subsequent 2 years^23,26^. For LDL-C, an increase was observed for the control group during follow-up, contributing considerably to the between-group difference at 3 years, especially among patients with ACS as the qualifying event. Analysis of adherence to lipid-lowering medication in this subgroup showed that discontinuation at some point during follow-up was common in both treatment groups, and that adverse symptoms with a non-compelling relationship to treatment was the dominating cause. In the control group, this was also the most common reason for permanent discontinuation^31^.

The possibility to adjust pharmacological treatment has been an integrated part of many intervention programmes with an effect on blood pressure and/or LDL-C levels^15,17,32^, though there are also examples in which medical prescribing has been recommended but not executed by the study team^18,19,33^. In INSPiRE-TMS, a GP or neurologist who was not part of the study team was responsible for prescribing in 6 out of 7 participating centres. This may have resulted in less effective management of pharmacological treatment and explain the more modest effect on risk factor levels in the InSPiRE-TMS study^10^.

It is reasonable to conclude the following: (1) the NAILED intervention effect on cardiovascular event recurrence was mediated through improved control of cardiovascular risk factors, and (2) the structured and repeated assessment of risk factor levels with prompt follow-up of pharmacological treatment contributed considerably to this effect. The latter included follow-up of adherence, re-challenge of treatment if non-compelling side-effects, switch to an alternative agent when needed, and enhancement of treatment through dose titration and/or addition of agents when indicated.

### Implications

From a health economics point of view, it is crucial to optimise preventive strategies so that as much prevention as possible can be achieved with minimal resources. Keeping follow-up intensity and commitments to a minimum may also be favourable to maximise patient participation and endurance over time, especially in high risk groups such as those with lower socioeconomic position^34^. The intervention evaluated in the NAILED trial represents a structured but simple form of long-term, secondary preventive follow-up designed to be broadly implemented on the population level. In this context, NAILED-CV makes a unique contribution to the evidence base, as previous studies have generally evaluated more resource-demanding interventions.

To put the NAILED-CV results into perspective, it is worth noting that the relative risk reduction achieved in our study is comparable to the gain of adding the newly introduced PCSK9 inhibitors to statin treatment, and the absolute risk reduction is considerably higher^35,36^. Though the preventive contribution of expensive agents, such as PCSK9 inhibitors, may be a valuable option in certain groups of patients, our results emphasise that, at the population level, there is still more to be gained by optimisation of already established and considerably cheaper alternatives. To further address the clinical implication of the NAILED intervention effect, a future cost effectivity analysis is needed and planned for.

### Strengths and limitations

The major strengths of NAILED-CV lie in the relatively unselected study sample, the large sample size, the long-term follow-up, and the blinded review of outcome events. The NAILED-CV study was a pragmatic trial striving to include all patients with a potential gain from optimised secondary preventive treatment. Exclusion criteria were kept to a minimum. In addition, the participation rate among eligible patients was high and few were lost to follow-up. Thus, the results are representative of what could be achieved by this intervention if implemented in clinical practice, at least in countries with similar populations and health care systems. The relatively large sample size enabled evaluation of the intervention effect on cardiovascular events, which is crucial for subsequent cost effectivity analysis. However, the study sample was too small to make firm conclusions on the subgroup level or regarding the effect on secondary endpoints, which is a limitation of the study. We followed and validated outcome events through overlapping sources, including the patients’ medical records, hospital registers, and death certificates. This created high sensitivity for identifying potential events, and any potential bias in validation based on treatment group allocation was avoided through blinded assessment. Although hospitalisation was generally indicated in all event classes included in this trial, we cannot rule out that occasional events were treated solely within primary care or that patients abstained medical contact in cases in which symptoms were perceived by the patient as mild or transient. Also, any event resulting in hospitalisation outside of the county and without subsequent transfer to Östersund Hospital would have been missed. Finally, blinding of study group allocation is not possible in this type of intervention study, and it is possible that the treatment of the control group was affected by study participation and, thus, deviated from the intended “usual care”. Although contacts with the control group were kept to a minimum, the yearly telephone interview for data collection may have improved adherence to pharmacological treatment, heightened awareness of risk factors, and affected life style behaviour. Furthermore, GPs were supplied with the results of blood pressure and blood lipid assessments, which may have resulted in actions that would not have been taken otherwise. However, such interference with usual care should primarily have resulted in underestimation of the intervention effect.

## Conclusion

Enhanced secondary preventive follow-up through yearly telephone contacts with a nurse for assessment and discussion of modifiable risk factors and adjustment of pharmacological treatment resulted in a decreased incidence of major cardiovascular events during long-term follow-up. The results can reasonably be concluded to have been mediated through improved adherence to treatment and improved risk factor control. NAILED-CV is the first study to show that improvement in cardiovascular morbidity and mortality can be achieved through this relatively simple model of secondary preventive follow-up after stroke, TIA, or ACS.

## Supplementary Information

Supplementary Methods 1.

Supplementary Methods 2.

Supplementary Table 1.

Supplementary Table 2.

Research Protocol.

## Data Availability

As open access to individual-level data was not specified in the original application approved by the ethics committee, the underlying data is only available upon reasonable request. Please contact the corresponding author.

## Abbreviations

NAILED-CV: Nurse-based, age-independent intervention to limit evolution of disease—cardiovascular
TIA: Transient ischemic disease
ACS: Acute coronary syndrome
RCT: Randomised controlled trial
LDL-C: Low-density lipoprotein cholesterol
MI: Myocardial infarction
UA: Unstable angina
SAH: Subarachnoid hemorrhage
CABG: Coronary artery bypass grafting
PCI: Percutaneous coronary intervention
GP: General practinoner
ARR: Absolute risk reduction
NNT: Numbers needed to treat
IQR: Interquartile range
CI: Confidence interval
