# Supplementary material for: Nurse-based secondary preventive follow-up by telephone reduced recurrence of cardiovascular events: a randomised controlled trial
Source: Sci Rep. 2021 Aug 2;11:15628. doi: 10.1038/s41598-021-94892-0 (PMC8329238; doi:10.1038/s41598-021-94892-0)
Supplement: Supplementary file 2 — Supplementary Methods 2. [file 41598_2021_94892_MOESM2_ESM.docx]

Supplementary methods 2

**International Classification of Disease, 10th revision (ICD-10), diagnosis**

Discharge ICD diagnosis (primary and secondary) included in the search of the local in-patient register to identify potential outcome events at other hospital departments other than the internal medicine department.

| **ICD-10 code** | **Condition** |
| --- | --- |
| **I21** | **Acute myocardial infarction** |
| I21.1 |  |
| I21.2 |  |
| I21.3 |  |
| I21.4 |  |
| I21.4A |  |
| I21.4B |  |
| I21.4W |  |
| I21.4X |  |
| I21.9 |  |
| **I20** | **Angina pectoris** |
| I20.0 |  |
| I20.1 |  |
| I20.8 |  |
| I20.9 |  |
| **G45** | **Transient cerebral ischaemic attacks and related syndromes** |
| G45.0 |  |
| G45.1 |  |
| G45.2 |  |
| G45.3 |  |
| G45.4 |  |
| G45.8 |  |
| G45.9 |  |
| **G46** | **Vascular syndromes of the brain in cerebrovascular diseases** |
| G46.0 |  |
| G46.1 |  |
| G46.2 |  |
| G46.3 |  |
| G46.4 |  |
| G46.5 |  |
| G46.6 |  |
| G46.7 |  |
| G46.8 |  |
| **G81** | **Hemiplegia** |
| G81.0 |  |
| G81.1 |  |
| G81.9 |  |
| **I60** | **Subarachnoid haemorrhage** |
| I60.0 |  |
| I60.1 |  |
| I60.2 |  |
| I60.3 |  |
| I60.4 |  |
| I60.5 |  |
| I60.6 |  |
| I60.7 |  |
| I60.8 |  |
| I60.9 |  |
| **I61** | **Intracerebral haemorrhage** |
| I61.0 |  |
| I61.1 |  |
| I61.2 |  |
| I61.3 |  |
| I61.4 |  |
| I61.5 |  |
| I61.6 |  |
| I61.8 |  |
| I61.9 |  |
| **I62** | **Other non-traumatic intracranial haemorrhage** |
| I62.0 |  |
| I62.1 |  |
| I62.9 |  |
| **I63** | **Cerebral infarction** |
| I63.0 |  |
| I63.1 |  |
| I63.2 |  |
| I63.3 |  |
| I63.4 |  |
| I63.5 |  |
| I63.6 |  |
| I63.8 |  |
| I63.9 |  |
| **I64** | **Stroke, not specified as haemorrhage or infarction** |
| I64.9 |  |
| **I65** | **Occlusion and stenosis of precerebral arteries, not resulting in cerebral infarction** |
| I65.0 |  |
| I65.1 |  |
| I65.2 |  |
| I65.3 |  |
| I65.8 |  |
| I65.9 |  |
| **I66** | **Occlusion and stenosis of cerebral arteries, not resulting in cerebral infarction** |
| I66.0 |  |
| I66.1 |  |
| I66.2 |  |
| I66.3 |  |
| I66.4 |  |
| I66.8 |  |
| I66.9 |  |
| **I67** | **Other cerebrovascular diseases** |
| I67.0 |  |
| I67.1 |  |
| I67.2 |  |
| I67.3 |  |
| I67.4 |  |
| I67.5 |  |
| I67.6 |  |
| I67.7 |  |
| I67.8 |  |
| I67.9 |  |
| **I68** | **Cerebrovascular disorders in diseases classified elsewhere** |
| I68.0 |  |
| I68.1 |  |
| I68.2 |  |
| I68.8 |  |
| **I69** | **Sequelae of cerebrovascular disease** |
| I69.0 |  |
| I69.0A |  |
| I69.0B |  |
| I69.1 |  |
| I69.2 |  |
| I69.3 |  |
| I69.4 |  |
| I69.8 |  |
