# Supplementary material for: Nurse-based secondary preventive follow-up by telephone reduced recurrence of cardiovascular events: a randomised controlled trial
Source: Sci Rep. 2021 Aug 2;11:15628. doi: 10.1038/s41598-021-94892-0 (PMC8329238; doi:10.1038/s41598-021-94892-0)
Supplement: Supplementary file 3 — Supplementary Table 1. [file 41598_2021_94892_MOESM3_ESM.docx]

**Supplementary table 1. Comparison of baseline characteristics for included patients, excluded patients, and patients who declined participation.**

|  | **Included** | **Excluded** | **Declined participation** |
| --- | --- | --- | --- |
| N (%) | 1890 (62·8) | 737 (24·5) | 384 (12·8) |
| Women | 689 (36·5) | 350 (47·5)*** | 187 (48·7)*** |
| Age, years | 71·0 (63·1-78·7) | 82·4 (74·3-87·6)*** | 81·2 (69·9-86·6)*** |
| Low education level | 947/1885 (50·2) | 511/659 (77·5)*** | 292/365 (80·0)*** |
| BMI^†^ | 26·6 (24·0-29·6) | 24·6 (21·8-27·8)*** | 25·7 (23·0-28·6)*** |
| eGFR, ml/min^‡^ | 81·4 (66·2-91·8) | 70·3 (52·9-83·5)*** | 70·8 (55·5-84·6)*** |
| Qualifying event |  |  |  |
| STEMI | 296 (15·7) | 53 (7·2) | 43 (11·2) |
| NSTEMI | 629 (33·3) | 136 (18·5)* | 110 (28·6) |
| Unstable angina | 95 (5·0) | 6 (0·8)*** | 9 (2·3)** |
| Ischaemic stroke | 508 (26·9) | 438 (59·4)*** | 143 (37·2) |
| Haemorrhagic stroke | 30 (1·6) | 52 (7·1)*** | 17 (4·4)** |
| TIA | 332 (17·6) | 51 (6·9)*** | 62 (16·1) |
| mRS>2 | 137/1884 (7·3) | 499/728 (68·5)*** | 88/383 (23·0)*** |
| Current/former smoker | 1078/1889 (57·1) | 313/702 (44·6)*** | 203/382 (53·1) |
| Atrial fibrillation | 299 (15·8) | 243 (33·0)*** | 106 (27·6)*** |
| Ischaemic heart disease | 326 (17·2) | 167/736 (22·7)*** | 84/383 (21·9)** |
| Peripheral artery disease | 45 (2·4) | 27/736 (3·7) | 9 (2·3) |
| Diabetes | 350 (18·5) | 174/736 (23·6)** | 90 (23·4)** |
| CKD (GFR<60 ml/min) | 342/1878 (18·2) | 257/732 (35·1)*** | 114/380 (30·0)*** |
| Congestive heart failure | 73 (3·9) | 98/736 (13·3)*** | 33 (8·6)*** |
| Hypertension | 1088 (57·6) | 511/736 (69·4)*** | 249 (64·8)** |
| Previous stroke | 170 (9·0) | 174 (23·6)*** | 57 (14·8)*** |
| Previous TIA | 69 (3·7) | 34 (4·6) | 19 (4·9) |
| Antihypertensive treatment | 1658 (87·7) | 616 (83·6)** | 335 (87·2) |
| 1 drug | 414 (21·9) | 176 (23·9) | 92 (24·0) |
| 2 drugs | 677 (35·8) | 244 (33·1) | 119 (31·0) |
| ≥3 drugs | 567 (30·0) | 196 (26·6) | 124 (32·3) |
| Lipid-lowering agent | 1600 (84·7) | 338 (45·9)*** | 251 (65·4)*** |
| Antiplatelet drug | 1678 (88·8) | 569 (77·2)*** | 311 (81·0)** |
| Warfarin | 212 (11·2) | 82 (11·1) | 62 (16·1) |

Data are given as n (%) or median (interquartile range). BMI, body mass index; eGFR, estimated glomerular filtration rate; CKD, chronic kidney dysfunction; TIA, transient ischaemic attack.

†Missing 3, 33, and 18 values for included, excluded, and declining patients, respectively.

‡ Missing 12, 5, and 4 values for included, excluded, and declining patients, respectively.

For categorical variables with missing values, the valid number of cases (denominator) is given separately for each variable.

Excluded patients and patients who declined participation were compared to the included group and significant differences indicated as follows: * p<0·05, **p<0·01, ***p<0·001.
