# Supplementary material for: Nurse-based secondary preventive follow-up by telephone reduced recurrence of cardiovascular events: a randomised controlled trial
Source: Sci Rep. 2021 Aug 2;11:15628. doi: 10.1038/s41598-021-94892-0 (PMC8329238; doi:10.1038/s41598-021-94892-0)
Supplement: Supplementary file 4 — Supplementary Table 2. [file 41598_2021_94892_MOESM4_ESM.docx]

**Supplementary table 2. Subgroup analysis of secondary endpoints**

|  | **All-cause mortality** | **Cardiovascular death** | **Myocardial infarction** | **Cardiac revascularisation** | **Stroke** | **TIA** |
| --- | --- | --- | --- | --- | --- | --- |
| **Intervention group** |  |  |  |  |  |  |
| Myocardial infarction/unstable angina | 98 (19·1) | 44 (8·6) | 41 (8·0) | 53 (10·4) | 26 (5·1) | 8 (1·6) |
| Stroke/TIA | 113 (26·2) | 44 (10·2) | 16 (3·7) | 17 (3·9) | 52 (12·0)^**^ | 23 (5·3) |
| Women | 85 (24·6) | 37 (10·7) | 18 (5·2) | 16 (4·6)^*^ | 37 (10·7) | 13 (3·8) |
| Men | 126 (21·1) | 51 (8·5) | 39 (6·5) | 54 (9·0) | 41 (6·9) | 18 (3·0) |
|  |  |  |  |  |  |  |
| **Control group** |  |  |  |  |  |  |
| Myocardial infarction/unstable angina | 114 (22·4) | 60 (11·8) | 55 (10·8) | 61 (12·0) | 25 (4·9) | 9 (1·8) |
| Stroke/TIA | 106 (24·2) | 45 (10·3) | 18 (4·1) | 18 (4·1) | 76 (17·4)^**^ | 27 (6·2) |
| Women | 91 (26·5) | 40 (11·7) | 29 (8·5) | 29 (8·5)^*^ | 44 (12·8) | 13 (3·8) |
| Men | 129 (21·4) | 65 (10·8) | 44 (7·3) | 50 (8·3) | 57 (9·5) | 23 (3·8) |

Data are given as N (%).

^*^ Significant interaction between sex and randomised allocation group (p=0·048), with significantly lower occurrence of cardiac revascularisation among women in the intervention group compared to women in the control group (HR 0·53, 95% CI 0·288-0·975, p=0·041).

^**^For participants with stroke or TIA as the qualifying event, the occurrence of a new stroke event was significantly lower in the intervention group than the control group (HR 0·69, 95% CI 0·46-0·98, p=0·036), but the interaction between qualifying event and randomised allocation group was not significant (p=0·253).
