# Supplementary material for: Nurse-based secondary preventive follow-up by telephone reduced recurrence of cardiovascular events: a randomised controlled trial
Source: Sci Rep. 2021 Aug 2;11:15628. doi: 10.1038/s41598-021-94892-0 (PMC8329238; doi:10.1038/s41598-021-94892-0)
Supplement: Supplementary file 5 — Research Protocol. [file 41598_2021_94892_MOESM5_ESM.docx]

**Research Protocol**

**General information**

Title:

**Secondary preventive follow-up after acute coronary syndrome (ACS) and stroke: nurse-led follow-up vs follow-up in primary healthcare - a randomized controlled study**

Principal investigator:

Thomas Mooe,

Senior professor, Department of Public Health and Clinical Medicine, Östersund, Umeå University, Umeå, Sweden

Responsible authority:

Region Jämtland Härjedalen

Box 654, 831 27 Östersund, Sweden

+46 063 153000

Affiliated academic center

Umeå University, 901 87 Umeå, Sweden

+46 090 7865000

Investigation site:

Östersund Hospital, 83183 Östersund, Sweden

+46 063 153000

Funders:

1. Unit of Research, Development and Education, Region Jämtland Härjedalen, 83127 Östersund, Sweden. +46 063-147500
2. Swedish Heart-Lung Foundation, Box 2167, 103 14 Stockholm, Sweden. +46 08 56624200

**Protocol revisions**

During the course of the trial the original research protocol was revised on several occasions. Revisions concerning the current study (“Prognosis study”) are specified below. Revisions relevant to the other sub studies are specified in the respective publications.

- Inclusion period and study sample

To enable sub studies of certain risk groups of interest, such as patients with diabetes and chronic kidney disease, the inclusion period was extended from 31 Dec 2011 to 31 Dec 2014. This resulted in a larger study sample than originally planned for.

- Duration of the project

As a consequence of the extended inclusion period, follow-up was prolonged until 31 Dec 2017.

- Timing of the first follow-up occasion

Changed from 6-10 week to 1 month after hospital discharge.

- Outcome

Cardiac revascularization was added as a component of the composite primary endpoint.

Please note that this document presents the original research protocol, without the above mention revisions.

**Project summary**

Backgound

Acute myocardial infarction (AMI) and stroke are the two most common causes of acute hospital care in Sweden, accounting for approximately 50 000 acute hospitalizations a year. Recurrent events are common and associated with substantial morbidity and mortality. Effective secondary preventive treatment is available but under-used. Data from Sweden as well as other European countries show that compliance with medication is defective and many patients do not reach the recommended treatment target levels for risk factors such as blood pressure and blood lipids.

Aim

To evaluate the effect of systematic follow-up performed by nurses on morbidity/mortality, compliance with medication and target achievements regarding risk factors after AMI and stroke.

Methods and outcomes

The participants will be randomized to nurse-based follow-up or usual care follow-up within primary care. During two consecutive years, all patients admitted due to stroke or AMI will be approached. After exclusion of those unable to participate, for example due to severe disease, the study participants will comprise approximately 600 patients with AMI and 650 patients with stroke. The participants will be followed for up to 5 years counted from the first inclusion. The main outcome variables include 1) recurrent events of AMI or stroke and mortality, 2) compliance with medication, and 3) the proportion who reach the target values for blood pressure and blood lipids.

Expected implication

The mechanisms explaining the insufficiency in secondary preventive care are not completely understood. Many factors may play a role such as prioritization and under manning within healthcare, multiple care givers and logistical problems. This study will offer an opportunity to evaluate the importance of systematic, long-term follow-up through nurses, a method that will side-step many of the difficulties in the current organization of health care responsible for secondary preventive follow-up in Sweden. A positive outcome in terms of lower morbidity and mortality would motivate permanent implementation and spread of this follow-up model. The study will also investigate factors that may affect compliance with preventive medication as well as target achievements regarding risk factors for recurrent cardiovascular events.

1. **Rationale and background information**

According to statistics from the National Board for Health and Welfare 2007 [1] the most common mortality cause in Sweden, for both women (42%), and men (41%), is cardiovascular diseases. In the group cardiovascular disorders, ischemic heart disease (including acute myocardial infarction, AMI) is responsible for 39% of the deaths in women and 50% in men. For stroke the respective percentages are 23% for women and 18% for men.

The incidence of AMI has decreased. Between the years 2001 and 2006 the standardized age incidence for men has decreased by 12% and for women by 7%.[2] AMI is however still one of the most common causes for acute hospitalization in Sweden, comprising approximately 25 200 patients during 2007. According to RIKS-STROKE 2007 the number of hospitalized cases for stroke is almost as many; 24 130 patients.[3]

Mortality due to myocardial infarction has decreased. Age standardized mortality due to myocardial infarction per 100 000 inhabitants has been reduced during the past 10-year period by an average of 4 % annually. AMI is still the single most common cause of mortality among women, 3 900 cases, and for men 4 900 cases. Corresponding number of deaths due to stroke (on all grounds) during 2007 for women was 4 600 and for men 3 300. There is also a decrease in incidence and mortality related to stroke.[4]

The reasons for the decrease in mortality following myocardial infarction is considered partly to be due to the lower incidence and partly to the ever improving efficiency in the treatment of myocardial infarction.[5] The fact that the incidence of myocardial infarction is seen to be decreasing can be accredited to a more favorable risk factor profile in the population.[4] The serious risk factors such as smoking, high blood pressure and high cholesterol, have improved during latter years. This is probably also the reason for the decreased incidence of stroke.

Secondary preventive measures contribute to improved risk factor profiles and the decreased incidence of myocardial infarction and stroke. Secondary prevention can also include measures to induce a favorable influence on smoking cessation, diet, physical activity, stress and psychosocial factors as well as encouraging the use of prescribed medication intended to reduce the risk of relapse. Effective secondary prevention can lead to a tangible reduction of mortality and relapse. Smoking cessation post myocardial infarction can e.g., after a year, lead to a relative risk reduction of 50 percent in mortality and relapse.[6]

Medication such as acetylsalicylic acid, statins and beta blockers following a myocardial infarction have been seen to lead to a relative risk reduction for both mortality and relapse to the extent of 20-30%.[7] A similar risk reduction for relapse and mortality has been noted when antiplatelet, antihypertensive and lipid-lowering medication is prescribed following a stroke.[8] Secondary preventive intervention is imperative for reducing mortality following a myocardial infarction, much more important than e.g. invasive treatment.[9]

May we then presume that current practical application of secondary preventive treatments following a myocardial infarction or stroke is effective? The answer is unfortunately negative from a national and international perspective. The EUROASPIRE overviews maintain that the risk factor impact in the aftermath of a myocardial infarction is highly unsatisfactory.[10] During the past 12 years, despite an increase in the use of prescribed secondary preventive medication, the percentage of patients achieving the set objectives regarding blood pressure, the number of diabetics with a satisfactory glycemic control and the number of patients that have stopped smoking have not improved. Lipid levels have improved but at the same time a tangible increase of obesity and diabetes has been noted. There is a substantial discrepancy between the standard promoted in guidelines and the actual result achieved in clinical practice.

The quality of secondary preventive care following a myocardial infarction in Sweden is evaluated in the SEPHIA register.[5] At a follow-up after one year the percentage of patients who had achieved the set objective regarding smoking cessation was 59%, systolic blood pressure 78%, low density lipoprotein cholesterol barely 70% and physical activity 43%. A summarized quality measure comprising 5 objectives was achieved by 19% of the patients after one year.

Regarding secondary prevention post stroke knowledge as to how the various treatments are applied is highly unsatisfactory. There is no systematic follow-up at either Swedish or European level, but a few reports regarding trials with more structured follow-up have been published.[11] A small project, including 328 patients from RIKS-STROKE revealed that only one third of the patients had achieved the set blood pressure objective after a 3 month follow-up. Other cardiovascular risk factors had been inadequately recorded and could not be evaluated.[12]

Accordingly, there is enormous potential for improved secondary preventive care, especially since available data overestimates the results of treatment. This is partly due to the fact that a substantial percentage of patients in the intended target group are not included in the reports and these patients usually attain poorer results. Age limits are applied, EUROASPIRE sets the age limit at 70 years of age and SEPHIA has an age limit of 75 years. This means that almost 50% of the myocardial infarction patients are excluded from the evaluations and it is often more difficult for older patients to achieve the risk factor objectives.[13]

Currently large drug trials are being implemented, each and every requiring huge investments with an expected absolute risk reduction regarding morbidity and mortality, at less than one percentage annually.[14, 15]. The potential profit of a more effective secondary prevention is much higher at a fraction of the cost.

Therefore, we plan to implement a randomized study regarding secondary prevention following a myocardial infarct or stroke, where the follow-up will be based on procedures led by specially trained nurses (intervention group) and compared with the current follow-up routines in Sweden, carried out in the primary healthcare (control group). Unique elements in the study are: 1) All patients with acute coronary syndrome (ACS) or stroke will be included, irrespective of age. Inclusion will be ongoing during the entire year, i.e. all patients with an ACS or stroke during the inclusion phase will be asked to participate. 2) Population based. All patients in the county of Jämtland will be included, i.e. those who are admitted to Östersund Hospital. 3) The nurses working with the intervention group must have adequate training and delegation to titrate statin doses. The contact with patients will be telephone-based. Focus will be on maximizing advantages and minimizing cost. 4) A long follow-up period. The majority of studies have a follow-up period of one or two years. For patients enrolled in the beginning of the inclusion period we plan for a follow-up of 5 years. The outcome will reflect a period of time relevant from the perspective of both patients and the healthcare system.

1. **Hypotheses**

Nurse-led, telephone-based secondary preventive follow-up after ACS or stroke, focusing on set objectives for lipids and blood pressure, smoking cessation and counselling/support regarding physical activity and diet, increase objective achievement, reduce morbidity/mortality and is more cost effective than traditional follow-up conducted in the primary healthcare.

1. **Study goals and objectives**

Compared with primary healthcare based secondary preventive follow-up after ACS and stroke, do nurse-based secondary preventive follow-up result in:

1. A more satisfactory prognosis regarding cardiovascular morbidity and overall mortality?
2. Improved compliance regarding the use of prescribed medication?
3. Better achievement of set objectives regarding cardiovascular risk factors?
4. A better cost efficiency?

Have the socioeconomic and stress related factors influenced:

1. The prognosis regarding cardiovascular morbidity and overall mortality?
2. Compliance regarding the use of prescribed medication?
3. Target achievement regarding cardiovascular factors?

### Study design

Open, randomized controlled trial with two (1:1) parallel groups.

### Methodology

**5.1 Definitions**

5.1.1 Definition ACS

1. Acute myocardial infarct type 1 according to the consensus document Circulation 2007 [16]
2. Unstable angina, typical ischemic symptoms in combination with dynamic ECG deviations (ST and/or T deviations) characteristic for ischemia.

5.1.2 Definition stroke

According to WHO: rapid development of clinical signs of focal (or global) disturbance of cerebral function with a duration surpassing 24 hours (if the process is not terminated due to mortality or surgery) and has no other explanation than vascular symptoms. A corresponding occurrence with duration less than 24 hours is defined as a transient ischemic attack (TIA).

5.1.3 Definition of intervention

Telephone-based monitoring and bloods after 6-10 weeks, 1, 2, 3, 4 and 5 years after an ACS respectively stroke/TIA, supervised by a nurse, who after specialized training can be delegated to titrate statin medication and modify antihypertensive treatment as instructed. Questions arising which are not covered by the standardized procedure are referred to a physician, who then contacts the patient if this is considered necessary. Modification of medication strives to achieve the desired objectives as soon as possible. Objective for blood pressure: seated systolic <130 – 140, seated diastolic <80 – 90 according to the revised guidelines stated by the European Society of Hypertension November 2009 (10). Objective for lipids: cholesterol <4.5, LDL-C <2.5. Counselling/support regarding smoking cessation and when necessary further contact via a smoking cessation clinic. Counselling/support regarding physical activity and diet. Bloods and diagnostic measurements are usually carried out at the nearest health center alternatively district nurse clinic according to common clinical routines.

5.1.4 Definition of control

Routine check-ups will be performed via the primary health care, to which the patient is referred when no further measures are called for at the coronary respectively stroke unit. In most cases this implies referral directly on discharge from hospital or after one or two initial follow-up appointments at the unit. This is consistent with the most common current routine in Sweden and the given times for reporting to on-going registration to the national quality resister SEPHIA. Risk factor objectives are assessed at each respective healthcare center.

**5.2 Inclusion criteria**

1. Acute myocardial infarct
2. Unstable angina according to the definition 5.1.1
3. Acute stroke or TIA according to the definition 5.1.2

**5.3 Exclusion criteria**

1. Mentally incapable of participation, due to dementia, mental illness, terminal illness.
2. Unable to communicate by telephone due to e. g. hearing disabilities or aphasia.
3. Patients with complicated illnesses requiring continued monitoring via specialists.
4. Patients with subarachnoid or subdural hemorrhage.

**5.4 Intervention**

According to definition 5.1.3

Randomization to the intervention or control group, respectively.

**5.5 Blood tests and examinations**

5.5.1 At inclusion routine samples during the initial hospital admission.

- Clinical Chemistry and Laboratory Medicine (Clin Chem Lab Med):

Routine hematology, Electrolyte status, high-sensitivity C-reactive protein (hs-CRP), lipids, fP-glucose, Hemogobin A1c (HbA1c)

- Physical measurement diagnostics:

Blood pressure (seated and standing), electrocardiography (ECG)

- Others:

Weight, height, abdominal circumference

Socioeconomic factors ([www.scb.se/sei](http://www.scb.se/sei))

Surveys pertaining to stress related factors according to Center for environmentally related illnesses and stress (CEOS).

Current medication.

5.5.2 After 6 – 10 weeks

- Clin Chem Lab Med:

Lipids, HbA1c – if previously atypical

- Physical measurement diagnostics:

Blood pressure (seated and standing)

- Others:

Weight

Current medication.

Number of health care contacts, hospital care, diagnosis, number of days in hospital, number of days on sick leave

Medication titration

Physical activity and smoking

5.5.3 After 1, 2, 3, 4 and 5 years

- Clin Chem Lab Med:

Lipids, HbA1c – if previously atypical

Physical measurement diagnostics:

Blood pressure (seated and standing), ECG

- Others:

Weight, abdominal circumference

Current medication and dosage

Hospital care, diagnosis, number of days in hospital

Medication titration

Physical activity and smoking

**5.6 Outcome variables**

5.6.1 Prognosis study

Primary: Mortality, relapse of myocardial infarct/stroke.

Secondary: Separate assessment of the individual components of the primary endpoint; hospital care: admittance and number of days in hospital.

5.6.2 Compliance study

Primary: Percentage of patients taking prescribed medication at the various times of follow-up (anti-platelet, anticoagulant, lipid-lowering and antihypertensive medication). Intervention group versus the control group.

Analysis of non-compliance according to Osterberg Blaschke.[17]

5.6.3 Study of risk factor objectives

Primary: Percentage of patients that achieved the set objectives at each respective time regarding lipids and blood pressure.

Secondary: Absolute values at each respective follow-up time regarding lipids and blood pressure. Intervention group versus the control group.

An analysis concerning the extent of the healthcare inputs necessary to achieve the set objectives: number of appointments, telephone contacts and titration occasions should be registered.

An analysis regarding the need of drugs to improve blood pressure and lipids: number of prescriptions, dosage, the necessity for more potent statins, all in relation to age and sex.

5.6.4 Cost-effective study

Primary: The cost per quality adjusted life year (QUALY).

Secondary: the actual cost per patient in each respective group.

5.6.5 Stress-related factors and prognosis

Primary: Morbidity and mortality in relation to variables in the surveys.

Secondary: Compliance and achieved objectives in relation to variables in the surveys.

5.6.6 Socioeconomics and prognosis

Primary: Morbidity and mortality in relation to the socioeconomics classification according to SEI ([www.scb.se/sei](http://www.scb.se/sei)).

Secondary: Compliance and achieved objectives in relation to SEI classification.

**5.7 Statistics**

5.7.1 Size of the study group

*5.7.1.1* Prognosis study primary end points

In a recently implemented study [18], with similar inclusion criteria regarding myocardial infarct (number of included subjects was 534) we found that the incidence of death, AMI and stroke at 2 years follow-up was approximately 50%. The expected frequency of these events will be around 60% after an average follow-up time of 3 – 4 years. A study comprising 300 patients in each respective group is then able to detect a risk reduction of 20 percentage point with 80% power and a significance level of 0.05.

A planned analysis, including both ACS and stroke patients, will consists of study groups comprising approximately 600 patients. A risk reduction of approximately 7- 8 percentage point can then be detected with 80% power and a significance level of 0.05.

5.7.2 Other analyses

*5.7.2.1* Primary end points

Parallel groups, estimation of relative risk with a 95% confidence interval and hazard ratio through Cox regression. Kaplan Meyer analysis with the log-rank test. Adjustment for baseline variables, if necessary, by a Cox regression model.

*5.7.2.2* Secondary end points

Please see 5.7.2.1

*5.7.2.3* Compliance analysis

At the various time points of follow-up, the percentage of patients in the intervention and control groups that follows respective ordinations; Chi-2 test. The Mann-Whitney test is used to compare the percentage of compliance regarding all cardiovascular medication for each patient in the intervention respectively control groups.

*5.7.2.4* Analysis of risk factor objectives

Primary, the percentage of patients who have achieved the set objectives: Chi2 test.

Secondary, absolute values: T-test, independent groups.

*5.7.2.5* Stress related factors

Dichotomized variables. Primary analysis as in 4.8.2.3

Secondary analysis regarding compliance and target values as in 4.8.2.3 respectively 4.8.2.4

*5.7.2.6* Socioeconomics analysis

Deployment of SEI-classes. Kaplan Meyer analysis regarding primary and secondary outcomes. Multivariate Cox regression analysis for identification of variables (including SEI-classes) that predict primary outcome. Chi2 test regarding percentage of compliance and percentage of achieved objectives.

### 6. Expected outcomes of the study

### 6.1 Prognosis study

Provides an opportunity to measure the outcome of serious incidents (mortality, stroke, myocardial infarct) directly between a follow-up routine based on specialist nurse supervision respectively current prevalent routines in primary healthcare. We know that secondary preventive measures are currently not optimal. The reasons for this are not entirely clear but factors such as staffing problems, numerous care givers being involved in the chain of decision-making, financial problems, overloading at all levels of the health care system, the need to prioritize, problems with logistics etc. all play their part in this. A favorable result of an organizationally straightforward nurse based secondary prevention with a lower outcome of morbidity and mortality would motivate implementation of this model as well as allowing for a permanent status. Significant positive effects regarding both health (morbidity and mortality) and economics would be foreseeable.

**6.2 Compliance**

Knowledge regarding adherence to prescribed medication and the reasons why prescriptions are not followed is highly inadequate. Available data show that approximately 50% of the patients have problems in following a given prescription within a period of 5 years. Most of the resulting problems could probably be solved and the effect of the treatment be retained. The study will show if access to and monitoring by a specialist nurse can enhance compliance and the effect of the treatment. The population aspect, i.e. the inclusion of all patients within a specific catchment area gives the study a unique strength regarding generalizability.

**6.3 Risk factor values**

Guidelines recommend target values regarding lipids and blood pressure following a myocardial infarct respectively stroke. The objectives are set according to results from large intervention studies. The target values for lipids have become more challenging and require significant measures to achieve. We have currently no reliable data that explains the prerequisites regarding patient contact, dosage titration, and adjustment of prescriptions etc. needed to achieve the current objectives. Knowledge pertaining to objectives and the necessary measures to achieve them are more or less non-existent regarding the group of older patients (>80 years of age) who dominate entirely regarding morbidity and mortality. The study will provide a solid bank of knowledge in this field.

**6.4 Cost effective study**

Provides a way to measure the financial effects of the intervention. It is measured partly by QUALYs and partly by directly measuring the actual cost alternatively saving per patient. Myocardial infarct and stroke are extremely costly for the healthcare system; consequently, a reduction of these incidences has the potential to save a substantial amount of money, which can be calculated accurately in the study.

**6.5 Stress related factors and prognosis**

Population based, prospective data showing stress related variable’s significance for morbidity and mortality following a myocardial infarct or stroke is not currently available.

**6.6 Socioeconomics and prognosis**

Knowledge about socioeconomic factors’ significance for morbidity, mortality, compliance and risk factor levels is highly inadequate, which has recently been commented by the National Board for Health and Welfare. A socioeconomic survey in the study provides grounds to improve this lack of knowledge.

### 7. Duration of the project

The study will be implemented during the period January 4^th^ 2010 through December 31^st^ 2014, with the inclusion period open until 31 dec 2011. ‘

**8. Data management**

Since all study measures, such as medication titration, are common treatment procedures, they will be documented as usual in the medical journal. The data source for laboratory data, blood pressure measurement data etc. is the electronic medical journal in use within the Jämtland Härjedalen county. Results will be transferred to a de-identified database in which each participant will be represented by a serial number. The database is located on a separate PC. The code list that links serial numbers with participants will be stored with the same level of confidentiality as other (paper) medical journal documents. Only those responsible for data registration and the principal investigator will have access to the code list and the PC. The part of the hospital building where the study documents are stored is looked with alarm function outside office hours. The code list will be destroyed in connection with the closure of the database at the end of the study. Thereafter it will not be possible to identify participants on the individual level. All data analyses will be performed on de-identified data.

**9. Safety considerations**

Since no other intervention than already well established secondary preventive treatments will be administered, no specific risk of harm has been identified other than those normally associated with the use of these treatments. In the intervention group, the participants may benefit from the long-term contact with a nurse. If the project turns out in favor of the nurse-based follow-up, all patients may gain from the study, given that the model is broadly implemented.

**10. Ethics**

For control group participants, participation will mean usual follow-up within primary health care. Intervention group participants will receive repeated telephone calls from a specially trained nurse in addition to the doctors’ appointments that are part of the standard care routine. In a two-year project, a similar follow-up routine was tested at the hospital cardiology department and no specific risks could be identified[18].

Written and oral information about the study will be given to potentially eligible patients by a study nurse before hospital discharge. The patients will thereafter be approached about participation and those who agree to participate will be asked to sign the written informed consent form.

**11. Dissemination of results and publication policy**

The study results will be documented as scientific manuscripts and published in scientific medical journals.

**12. References**

1. Socialstyrelsen. Dödsorsaker 2007. Socialstyrelsen 2009.

2. Socialstyrelsen. Hjärtinfarkter 1987-2006. Socialstyrelsen 2008.

3. Årsrapport Riks-Stroke 2007. Riks-Stroke 2008.

4. Socialstyrelsen. Folkhälsorapport 2009. Socialstyrelsen 2009.

5. Stenestrand U, Wallentin L, Lindahl B, Tyden P, Hambraeus K, James S, et al. Årsrapport 2007 - RIKS-HIA, SEPHIA och SCAAR. UCR, Uppsala.; 2008.

6. Wilhelmsson C, Vedin JA, Elmfeldt D, Tibblin G, Wilhelmsen L. Smoking and myocardial infarction. Lancet 1975;2:1157-60.

7. Rockson SG, deGoma EM, Fonarow GC. Reinforcing a continuum of care: in-hospital initiation of long-term secondary prevention following acute coronary syndromes. Cardiovasc Drugs Ther 2007;21(5):375-88.

8. Talelli P, Greenwood RJ. Recurrent stroke: where do we stand with the secondary prevention of noncardioembolic ischaemic strokes? Ther Adv Cardiovasc Dis 2008;2(5):387-405.

9. Bjorck L, Rosengren A, Bennett K, Lappas G, Capewell S. Modelling the decreasing coronary heart disease mortality in Sweden between 1986 and 2002. Eur Heart J 2009;30(9):1046-56.

10. Kotseva K, Wood D, De Backer G, De Bacquer D, Pyorala K, Keil U. Cardiovascular prevention guidelines in daily practice: a comparison of EUROASPIRE I, II, and III surveys in eight European countries. Lancet 2009;373(9667):929-40.

11. Bushnell C, Zimmer L, Schwamm L, Goldstein LB, Clapp-Channing N, Harding T, et al. The Adherence eValuation After Ischemic Stroke Longitudinal (AVAIL) registry: design, rationale, and baseline patient characteristics. Am Heart J 2009;157(3):428-435 e2.

12. Collen A-C, Lagerlöf A, Nieburg I, Carlberg B. Blodtryck efter stroke - hur många når mål? Svenska Läkaresällskapets Riksstämma 2008;Internmedicin:6P.

13. Hanna IR, Wenger NK. Secondary prevention of coronary heart disease in elderly patients. Am Fam Physician 2005;71(12):2289-96.

14. Barter PJ, Caulfield M, Eriksson M, Grundy SM, Kastelein JJ, Komajda M, et al. Effects of torcetrapib in patients at high risk for coronary events. N Engl J Med 2007;357(21):2109-22.

15. Bhatt DL, Fox KA, Hacke W, Berger PB, Black HR, Boden WE, et al. Clopidogrel and aspirin versus aspirin alone for the prevention of atherothrombotic events. N Engl J Med 2006;354(16):1706-17.

16. Thygesen K, Alpert JS, White HD, Jaffe AS, Apple FS, Galvani M, et al. Universal definition of myocardial infarction. Circulation 2007;116(22):2634-53.

17. Osterberg L, Blaschke T. Adherence to medication. N Engl J Med 2005;353(5):487-97.

18. Ohlsson-Önerud Å, Mooe T, Modica A, Friberg B, Persson K. KAPRIS - ett Kardiovaskulärt Prevention och Risk Intervenerande System. Läkaresällskapets Riksstämma 2008;Internmedicin:17P.
